# Supplementary material for: Discovery and application of insertion-deletion (INDEL) polymorphisms for QTL mapping of early life-history traits in Atlantic salmon
Source: BMC Genomics. 2010 Mar 8;11:156. doi: 10.1186/1471-2164-11-156 (PMC2838853; doi:10.1186/1471-2164-11-156)
Supplement: Additional file 2 — Information on developed 76 locus single-run INDEL panel in Atlantic salmon. Information on fluorescence labeling, primer concentrations, PCR pooling and links to alignments, INDEL motifs and GENESCAN (Burge and Karlin 1997) predictions of genes/exons are available in html format. [file 1471-2164-11-156-S2.ZIP › Additionalfile2/table10585.html]

Contig Alignment

```
Base	A B C D E   
1	G . . . .    
2	G . . . .    
3	G . . . .    
4	G . . . .    
5	T . . . .    
6	A . . . .    
7	T . . . .    
8	T . . . .    
9	T . . . .    
10	G . . . .    
11	C . . . .    
12	T . . . .    
13	G . . . .    
14	T . . . .    
15	A . . . .    
16	C . . . .    
17	A . . . .    
18	T . . . .    
19	T . . . .    
20	T . . . .    
21	C . . . .    
22	T . . . .    
23	A . . . .    
24	C . . . .    
25	C . . . .    
26	G . . . .    
27	T . . . .    
28	T . . . .    
29	C . . . .    
30	C . . . .    
31	C . . . .    
32	T . . . .    
33	T . . . .    
34	G . . . .    
35	T . . . .    
36	C . . . .    
37	A . . . .    
38	G . . . .    
39	C . . . .    
40	C . . . .    
41	A . . . .    
42	C . . . .    
43	A . . . .    
44	A . . . .    
45	G . . . .    
46	A . . . .    
47	C . . . .    
48	A . . . .    
49	G . . . .    
50	A . . . .    
51	C . . . .    
52	A . . . .    
53	G . . . .    
54	G . . . .    
55	C . . . .    
56	A . . . .    
57	G . . . .    
58	T . . . .    
59	T . . . .    
60	C . . . .    
61	C . . . .    
62	A . . . .    
63	G . . . .    
64	G . . . .    
65	A . . . .    
66	G . . . .    
67	C . . . .    
68	T . . . .    
69	G . . . .    
70	G . . . .    
71	A . . . .    
72	G . . . .    
73	G . . . .    
74	A . . . .    
75	G . . . .    
76	C . . . .    
77	T . . . .    
78	A . . . .    
79	T . . . .    
80	C . . . .    
81	C . . . .    
82	A . . . .    
83	A . . . .    
84	C . . . .    
85	C . . . .    
86	T . . . .    
87	G . . . .    
88	A . . . .    
89	C . . . .    
90	C . . . .    
91	A . . . .    
92	G . . . .    
93	G . . . .    
94	C . . . .    
95	T . . . .    
96	G . . . .    
97	C . . . .    
98	A . . . .    
99	G . . . .    
100	A . . . .    
101	A . . . .    
102	T . . . .    
103	G . . . .    
104	A . . . .    
105	G . . . .    
106	C . . . .    
107	T . . . .    
108	G . . . .    
109	G . . . .    
110	A . . . .    
111	C . . . .    
112	G . . . .    
113	G . . . .    
114	C . . . .    
115	A . . . .    
116	G . . . .    
117	A . . . .    
118	G . . . .    
119	G . . . .    
120	G . . . .    
121	G . . . .    
122	A . . . .    
123	G . . . .    
124	T . . . .    
125	C . . . .    
126	C . . . .    
127	C . . . .    
128	T . . . .    
129	C . . . .    
130	A . . . .    
131	T . . . .    
132	G . . . .    
133	C . . . .    
134	A . . . .    
135	A . . . .    
136	T . . . .    
137	C . . . .    
138	T . . . .    
139	C . . . .    
140	C . . . .    
141	C . . . .    
142	A . . . .    
143	G . . . .    
144	C . . . .    
145	T . . . .    
146	C . . . .    
147	T . . . .    
148	C . . . .    
149	A . . . .    
150	A . . . .    
151	T . . . .    
152	T . . . .    
153	C . . . .    
154	C . . . .    
155	C . . . .    
156	T . . . .    
157	T . . . .    
158	A . . . .    
159	A . . . .    
160	A . . . .    
161	G . . . .    
162	A . . . .    
163	C . . . .    
164	T . . . .    
165	G . . . .    
166	A . . . .    
167	C . . . .    
168	T . . . .    
169	A . . . .    
170	C . . . .    
171	T . . . .    
172	C . . . .    
173	T . . . .    
174	G . . . .    
175	A . . . .    
176	C . . . .    
177	A . . . .    
178	A . . . .    
179	G . . . .    
180	A . . . .    
181	T . . . .    
182	A . . . .    
183	G . . . .    
184	A . . . .    
185	T . . . .    
186	A . . . .    
187	T . . . .    
188	C . . . .    
189	T . . . .    
190	C . . . .    
191	T . . . .    
192	A . . . .    
193	C . . . .    
194	C . . . .    
195	A . . . .    
196	C . . . .    
197	G . . . .    
198	C . . . .    
199	C . . . .    
200	A . . . .    
201	A . . . .    
202	T . . . .    
203	A . . . .    
204	C . . . .    
205	T . . . .    
206	T . . . .    
207	T . . . .    
208	T . . . .    
209	G . . . .    
210	C . . . .    
211	C . . . .    
212	A . . . .    
213	T . . . .    
214	T . . . .    
215	A . . . .    
216	G . . . .    
217	T . . . .    
218	T . . . .    
219	T . . . .    
220	G . . . .    
221	A . . . .    
222	C . . . .    
223	A . . . .    
224	T . . . .    
225	T . . . .    
226	T . . . .    
227	C . . . .    
228	T . . . .    
229	A . . . .    
230	T . . . .    
231	T . . . .    
232	A . . . .    
233	A . . . .    
234	A . . . .    
235	A . . . .    
236	A . . . .    
237	C . . . .    
238	A . . . .    
239	C . . . .    
240	A . . . .    
241	T . . . .    
242	A . . . .    
243	A . . . .    
244	T . . . .    
245	T . . . .    
246	T . . . .    
247	A . . . .    
248	T . . . .    
249	A . . . .    
250	T . . . .    
251	G . . . .    
252	A . . . .    
253	A . . . .    
254	A . . . .    
255	G . . . .    
256	T . . . .    
257	G . . . .    
258	T . . . .    
259	T . . . .    
260	G . . . .    
261	T . . . .    
262	G . . . .    
263	C . . . .    
264	T . . . .    
265	A . . . .    
266	C . . . .    
267	T . . . .    
268	C . . . .    
269	A . . . .    
270	C . . . .    
271	T . . . .    
272	G . . . .    
273	T . . . .    
274	T . . . .    
275	T . . . .    
276	G . . . .    
277	A . . . .    
278	A . . . .    
279	A . . . .    
280	T . . . .    
281	G . . . .    
282	T . . . .    
283	T . . . .    
284	C . . . .    
285	A . . . .    
286	C . . . .    
287	G . . . .    
288	T . . . .    
289	T . . . .    
290	T . . . .    
291	C . . . .    
292	T . . . .    
293	A . . . .    
294	G . . . .    
295	A . . . .    
296	T . . . .    
297	T . . . .    
298	T . . . .    
299	T . . . .    
300	T . . . .    
301	T . . . .    
302	T . . . .    
303	A . . . .    
304	C . . . .    
305	T . . . .    
306	T . . . .    
307	A . A . .    
308	C . C . .    
309	A . A . .    
310	T . T . .    
311	A . A . .    
312	T . T . .    
313	A . A . .    
314	A . A . .    
315	C . C . .    
316	T . T . .    
317	G . G . .    
318	T . T . .    
319	A . A . .    
320	A . A . .    
321	A . A . .    
322	A . A . .    
323	C . C . .    
324	T . T . .    
325	A . A . .    
326	T . T . .    
327	G . G . .    
328	C . C . .    
329	C . C . .    
330	A . A . .    
331	C . C . .    
332	T . T . .    
333	T . T . .    
334	A . A . .    
335	A . A . .    
336	C . C . .    
337	G . G . .    
338	C . C . .    
339	A . A . .    
340	T . T . .    
341	G . G . .    
342	T . T . .    
343	A . A . .    
344	C . C . .    
345	A . A . .    
346	C . C . .    
347	A . A . .    
348	T . T . .    
349	T . T . .    
350	T . T . .    
351	T . T . .    
352	C . C . .    
353	A . A . .    
354	A . A . .    
355	C . C . .    
356	A . A . .    
357	G . G . .    
358	A . A . .    
359	A . A . .    
360	A . A . .    
361	A . A . .    
362	A . A . .    
363	A . A . .    
364	A . A . .    
365	C . C . .    
366	A . A . .    
367	C . C . .    
368	C . C . .    
369	A . A . .    
370	C . C . .    
371	T . T . .    
372	G . G . .    
373	T . T . .    
374	C . C . .    
375	C . C . .    
376	T . T . .    
377	A . A . .    
378	A . A . .    
379	T . T . .    
380	T . T . .    
381	G . G . .    
382	G . G . .    
383	C . C . .    
384	A . A . .    
385	C . C . .    
386	T . T . .    
387	T . T . .    
388	G . G . .    
389	G . G . .    
390	A . A . .    
391	G . G . .    
392	C . C . .    
393	A . A . .    
394	T . T . .    
395	T . T . .    
396	G . G . .    
397	T . T . .    
398	A . A . .    
399	T . T . .    
400	T . T . .    
401	G . G . .    
402	T . T . .    
403	G . G . .    
404	G . G . .    
405	A . A . .    
406	C . C . .    
407	A . A . .    
408	G . G . .    
409	T . T . .    
410	T . T . .    
411	T . T . .    
412	T . T . .    
413	T . T . .    
414	T . T . .    
415	T . T . .    
416	T . T . .    
417	T . T . .    
418	T . T . .    
419	T . T . .    
420	T . T . .    
421	T . T . .    
422	A A A . .    
423	A A A . .    
424	A A A . .    
425	T T T . .    
426	T T T . .    
427	C C C . .    
428	T T T . .    
429	A A A . .    
430	T T T . .    
431	T T T . .    
432	T T T . .    
433	A A A . .    
434	C C C . .    
435	C C C . .    
436	T T T . .    
437	T T T . .    
438	G G G . .    
439	C C C . .    
440	C C C . .    
441	G G G . .    
442	G G G . .    
443	T T T . .    
444	A A A . .    
445	A A A . .    
446	T T T . .    
447	A A A . .    
448	A A A . .    
449	T T T . .    
450	G G G . .    
451	C C C . .    
452	A A A . .    
453	T T T . .    
454	G G G . .    
455	G G G . .    
456	G G G . .    
457	G G G . .    
458	C C C . .    
459	T T T . .    
460	G G G G .    
461	A A A A .    
462	A A A A .    
463	G G G G .    
464	A A A A .    
465	G G G G .    
466	G G G G .    
467	G G G G .    
468	C C C C .    
469	T T T T .    
470	C C C C .    
471	T T T T .    
472	C C C C .    
473	A A A A .    
474	T T T T .    
475	T T T T .    
476	A A A A .    
477	A A A A .    
478	C C C C .    
479	A A A A .    
480	A A A A .    
481	T T T T .    
482	C C C C .    
483	T T T T .    
484	A A A A .    
485	T T T T .    
486	T T T T .    
487	G G G G .    
488	T T T T .    
489	G G G G .    
490	A A A A .    
491	C C C C .    
492	A A A A .    
493	A A A A .    
494	G G G G .    
495	A A A A .    
496	T T T T .    
497	G G G G .    
498	G G G G .    
499	C C C C .    
500	T T T T .    
501	T T T T .    
502	T T T T .    
503	C C C C .    
504	T T T T .    
505	T T T T .    
506	T T T T .    
507	A A A A .    
508	T T T T .    
509	T T T T .    
510	G G G G .    
511	T T T T .    
512	T T T T .    
513	A A A A .    
514	T T T T .    
515	G G G G .    
516	A A A A .    
517	G G G G .    
518	C C C C .    
519	A A A A .    
520	C C C C .    
521	C C C C .    
522	A A A A .    
523	C C C C .    
524	T T T T .    
525	G G G G .    
526	A A A A .    
527	A A A A .    
528	G G G G .    
529	G G G G .    
530	T T T T .    
531	C C C C .    
532	T T T T .    
533	T T T T .    
534	A A A A .    
535	T T T T .    
536	G G G G .    
537	G G G G .    
538	T T T T .    
539	G G G G .    
540	T T T T .    
541	T T T T .    
542	T T T T .    
543	T - T - .  2	 indel  
544	C - C - .  2	 indel  
545	A A A A .    
546	A A A A .    
547	G G G G .    
548	A A A A .    
549	C C C C .    
550	A A A A .    
551	A A A A .    
552	C C C C .    
553	T T T T .    
554	C C C C .    
555	T T T T .    
556	G G G G .    
557	A A A A .    
558	A A A A .    
559	C C C C .    
560	C C C C .    
561	T T T T .    
562	C C C C .    
563	G G G G .    
564	C C C C .    
565	A A A A .    
566	A A A A .    
567	G G G G .    
568	T T T T .    
569	T T T T .    
570	A A A A .    
571	A A A A .    
572	A A A A .    
573	A A A A .    
574	T T T T .    
575	T T T T .    
576	T T T T .    
577	G G G G .    
578	C C C C .    
579	G G G G .    
580	T T T T .    
581	A A A A .    
582	G G G G .    
583	A A A A .    
584	G G G G .    
585	C C C C .    
586	T T T T .    
587	T T T T .    
588	C C C C .    
589	C C C C .    
590	T T T T .    
591	A A A A .    
592	T T T T .    
593	T T T T .    
594	G G G G .    
595	G G G G .    
596	A A A A .    
597	C C C C .    
598	G G G G .    
599	A A A A .    
600	T T T T .    
601	T T T T .    
602	C C C C .    
603	C C C C .    
604	G G G G .    
605	T T T T .    
606	A A A A .    
607	T T T T .    
608	T T T T .    
609	C C C C .    
610	A A A A .    
611	A A A A .    
612	C C C C .    
613	C C C C .    
614	T T T T .    
615	A A A A .    
616	A A A A .    
617	T T T T .    
618	G G G G .    
619	T T T T .    
620	T T T T .    
621	A A A A .    
622	A A A A .    
623	G G G G .    
624	A A A A .    
625	T T T T .    
626	T T T T .    
627	T T T T .    
628	T T T T .    
629	T T T T .    
630	T T T T .    
631	G G G G .    
632	T T T T .    
633	T T T T .    
634	T T T T .    
635	T T T T .    
636	T T T T .    
637	A A A A .    
638	G G G G .    
639	A A A A .    
640	G G G G .    
641	T T T T .    
642	T T T T .    
643	T T T T .    
644	G G G G .    
645	T T T T .    
646	T T T T .    
647	T T T T .    
648	G G G G .    
649	A A A A .    
650	T T T T .    
651	A A A A .    
652	T T T T .    
653	T T T T .    
654	A A A A .    
655	A A A A .    
656	T T T T .    
657	A A A A .    
658	T T T T .    
659	G G G G .    
660	T T T T .    
661	A A A A .    
662	A A A A .    
663	T T T T .    
664	C C C C .    
665	A A A A .    
666	G G G G .    
667	C C C C .    
668	C C C C .    
669	T T T T .    
670	G G G G .    
671	C C C C .    
672	T T T T .    
673	T T T T .    
674	A A A A .    
675	A A A A .    
676	G G G G .    
677	T T T T .    
678	T T T T .    
679	A A A A .    
680	T T T T .    
681	T T T T .    
682	T T T T .    
683	G G G G .    
684	T T T T .    
685	A A A A .    
686	G G G G .    
687	T T T T .    
688	C C C C .    
689	C C C C .    
690	A A A A .    
691	A A A A .    
692	A A A A .    
693	T T T T .    
694	G G G G .    
695	T T T T .    
696	T T T T .    
697	A A A A .    
698	C C C C .    
699	T T T T .    
700	G G G G .    
701	C C C C .    
702	A A A A .    
703	T T T T .    
704	A A A A .    
705	A A A A .    
706	A A A A .    
707	C C C C .    
708	T T T T .    
709	C C C C .    
710	A A A A .    
711	A A A A .    
712	C C C C .    
713	A A A A .    
714	A A A A .    
715	A A A A .    
716	T T T T .    
717	A A A A .    
718	G G G G .    
719	T T T T .    
720	T T T T .    
721	T T T T .    
722	A A A A .    
723	C C C C .    
724	A A A A .    
725	T T T T .    
726	A A A A .    
727	G G G G .    
728	T T T T .    
729	. A A A .    
730	. T T T .    
731	. T T T .    
732	. G G G .    
733	. A A A .    
734	. A A A .    
735	. A A A .    
736	. T T T .    
737	. A A A .    
738	. A A A .    
739	. A A A .    
740	. C C C .    
741	. A A A .    
742	. T T T .    
743	. T T T .    
744	. T T T .    
745	. T T T .    
746	. G G G .    
747	. T T T .    
748	. A A A .    
749	. C C C .    
750	. C C C .    
751	. A A A .    
752	. T T T .    
753	. G G G .    
754	. G G G .    
755	. T T T .    
756	. T T T .    
757	. G G G .    
758	. T T T .    
759	. C C C .    
760	. T T T .    
761	. G G G .    
762	. A A A .    
763	. A A A .    
764	. C C C .    
765	. A A A .    
766	. A A A .    
767	. T T T .    
768	. G G G .    
769	. T T T .    
770	. T T T .    
771	. T T T .    
772	. T T T .    
773	. T T T .    
774	. G G G .    
775	. T T T .    
776	. G G G .    
777	. A A A .    
778	. C C C .    
779	. A A A .    
780	. T T T .    
781	. A A A .    
782	. G G G .    
783	. A A A .    
784	. A A A .    
785	. A A A .    
786	. A A A .    
787	. G G G .    
788	. C C C .    
789	. T T T .    
790	. A A A .    
791	. T T T .    
792	. T T T .    
793	. T T T .    
794	. T T T .    
795	. C C C .    
796	. A A A .    
797	. C C C .    
798	. T T T .    
799	. G G G .    
800	. C C C .    
801	. T T T .    
802	. G G G .    
803	. C C C .    
804	. T T T .    
805	. T T T .    
806	. A A A .    
807	. A A A .    
808	. A A A .    
809	. C C C .    
810	. A A A .    
811	. G G G .    
812	. T T T .    
813	. G G G .    
814	. A A A .    
815	. A A A .    
816	. T T T .    
817	. A A A .    
818	. C C C .    
819	. A A A .    
820	. G G G .    
821	. A A A .    
822	. A A A .    
823	. A A A .    
824	. T T T .    
825	. G G G .    
826	. A A A .    
827	. A A A .    
828	. C C C .    
829	. T T T .    
830	. G G G .    
831	. C C C .    
832	. A A A .    
833	. C C C .    
834	. A A A .    
835	. T T T .    
836	. A A A .    
837	. T T T .    
838	. T T T .    
839	. C C C .    
840	. T T T .    
841	. T T T .    
842	. T T T .    
843	. C C C .    
844	. T T T .    
845	. C C C .    
846	. A A A .    
847	. A A A .    
848	. C C C .    
849	. A A A .    
850	. C C C .    
851	. A A A .    
852	. A A A .    
853	. T T T .    
854	. T T T .    
855	. T T T .    
856	. G G G .    
857	. C C C .    
858	. T T T .    
859	. T T T .    
860	. C C C .    
861	. G G G .    
862	. T T T .    
863	. A A A .    
864	. A A A .    
865	. A A A .    
866	. A A A .    
867	. G G G .    
868	. G G G .    
869	. T T T .    
870	. G G G .    
871	. T T T .    
872	. T T T .    
873	. A A A .    
874	. T T T .    
875	. G G G .    
876	. T T T .    
877	. T T T .    
878	. G G G .    
879	. C C C .    
880	. T T T .    
881	. T T T .    
882	. T T T .    
883	. C C C .    
884	. A A A .    
885	. T T T .    
886	. T T T .    
887	. T T T .    
888	. C C C .    
889	. C C C .    
890	. T T T T    
891	. G G G G    
892	. T T T T    
893	. C C C C    
894	. A A A A    
895	. A A A A    
896	. A A A A    
897	. A A A A    
898	. G G G G    
899	. T T T T    
900	. G G G G    
901	. C C C C    
902	. A A A A    
903	. C C C C    
904	. C C C C    
905	. C C C C    
906	. T T T T    
907	. C C C C    
908	. T T T T    
909	. G G G G    
910	. A A A A    
911	. C C C C    
912	. T T T T    
913	. T T T T    
914	. G G G G    
915	. T T T T    
916	. T T T T    
917	. T T T T    
918	. G G G G    
919	. T T T T    
920	. A A A A    
921	. C C C C    
922	. A A A A    
923	. C C C C    
924	. T T T T    
925	. G G G G    
926	. A A A A    
927	. G G G G    
928	. T T T T    
929	. T T T T    
930	. G G G G    
931	. G G G G    
932	. T T T T    
933	. A A A A    
934	. A A A A    
935	. A A A A    
936	. T T T T    
937	. A A A A    
938	. T T T T    
939	. A A A A    
940	. G G G G    
941	. A A A A    
942	. A A A A    
943	. A A A A    
944	. A A A A    
945	. G G G G    
946	. T T T T    
947	. T T T T    
948	. T T T T    
949	. G G G G    
950	. G G G G    
951	. T T T T    
952	. C C C C    
953	. A A A A    
954	. T T T T    
955	. A A A A    
956	. C C C C    
957	. G G G G    
958	. C C C C    
959	. A A A A    
960	. C C C C    
961	. A A A A    
962	. T T T T    
963	. C C C C    
964	. C C C C    
965	. T T T T    
966	. T T T T    
967	. A A A A    
968	. A A A A    
969	. A A A A    
970	. C C C C    
971	. C C C C    
972	. A A A A    
973	. T T T T    
974	. G G G G    
975	. T T T T    
976	. A A A A    
977	. A A A A    
978	. C C C C    
979	. C C C C    
980	. A A A A    
981	. T T T T    
982	. T T T T    
983	. T T T T    
984	. G G G G    
985	. C C C C    
986	. T T T T    
987	. T T T T    
988	. T T T T    
989	. T T T T    
990	. T T T T    
991	. G G G G    
992	. T T T T    
993	. T T T T    
994	. T T T T    
995	. T T T T    
996	. G G G G    
997	. C C C C    
998	. T T T T    
999	. T T T T    
1000	. C C C C    
1001	. T T T T    
1002	. A A A A    
1003	. G G G G    
1004	. G G G G    
1005	. A A A A    
1006	. G T T T    
1007	. . A A A    
1008	. . A A A    
1009	. . T A A    
1010	. . T T T    
1011	. . T T T    
1012	. . T T T    
1013	. . T T T    
1014	. . T T T    
1015	. . T T T    
1016	. . T T T    
1017	. . A A A    
1018	. . A A A    
1019	. . T T T    
1020	. . A A A    
1021	. . C C C    
1022	. . C C C    
1023	. . T T T    
1024	. . C C C    
1025	. . A A A    
1026	. . A A A    
1027	. . A A A    
1028	. . T T T    
1029	. . T T T    
1030	. . G G G    
1031	. . C C C    
1032	. . C C C    
1033	. . A A A    
1034	. . T T T    
1035	. . T T T    
1036	. . G G G    
1037	. . G G G    
1038	. . G G G    
1039	. . G G G    
1040	. . A A A    
1041	. . A A A    
1042	. . T T T    
1043	. . G G G    
1044	. . A A A    
1045	. . T T T    
1046	. . A A A    
1047	. . G G G    
1048	. . A A A    
1049	. . C C C    
1050	. . A A A    
1051	. . G G G    
1052	. . A A A    
1053	. . T T T    
1054	. . G G G    
1055	. . G G G    
1056	. . C C C    
1057	. . G G G    
1058	. . C C C    
1059	. . T T T    
1060	. . G G G    
1061	. . T T T    
1062	. . G G G    
1063	. . A A A    
1064	. . C C C    
1065	. . C C C    
1066	. . T T T    
1067	. . C C C    
1068	. . A A A    
1069	. . T T T    
1070	. . T T T    
1071	. . A A A    
1072	. . C C C    
1073	. . A A A    
1074	. . A A A    
1075	. . G G G    
1076	. . T T T    
1077	. . C C C    
1078	. . T T T    
1079	. . G G G    
1080	. . A A A    
1081	. . T T T    
1082	. . C C C    
1083	. . A A A    
1084	. . T T T    
1085	. . G G G    
1086	. . T T T    
1087	. . T T T    
1088	. . C C C    
1089	. . A A A    
1090	. . G G G    
1091	. . T T T    
1092	. . G G G    
1093	. . G G G    
1094	. . T T T    
1095	. . C C C    
1096	. . A C C    
1097	. . . T T    
1098	. . . G G    
1099	. . . T T    
1100	. . . T T    
1101	. . . T T    
1102	. . . C C    
1103	. . . A A    
1104	. . . G G    
1105	. . . A A    
1106	. . . G G    
1107	. . . A A    
1108	. . . A A    
1109	. . . G G    
1110	. . . T T    
1111	. . . A A    
1112	. . . A A    
1113	. . . A A    
1114	. . . G G    
1115	. . . T T    
1116	. . . T T    
1117	. . . A A    
1118	. . . C C    
1119	. . . T T    
1120	. . . A A    
1121	. . . A A    
1122	. . . G G    
1123	. . . A A    
1124	. . . T T    
1125	. . . G G    
1126	. . . T T    
1127	. . . T T    
1128	. . . . A    
1129	. . . . C    
1130	. . . . T    
1131	. . . . C    
1132	. . . . A    
1133	. . . . T    
1134	. . . . G    
1135	. . . . A    
1136	. . . . T    
1137	. . . . G    
1138	. . . . T    
1139	. . . . T    
1140	. . . . G    
1141	. . . . T    
1142	. . . . G    
1143	. . . . C    
1144	. . . . T    
1145	. . . . C    
1146	. . . . T    
1147	. . . . C    
1148	. . . . T    
1149	. . . . T    
1150	. . . . T    
1151	. . . . A    
1152	. . . . T    
1153	. . . . T    
1154	. . . . T    
1155	. . . . G    
1156	. . . . T    
1157	. . . . T    
1158	. . . . A    
1159	. . . . A    
1160	. . . . A    
1161	. . . . T    
1162	. . . . T    
1163	. . . . A    
1164	. . . . T    
1165	. . . . G    
1166	. . . . G    
1167	. . . . C    
1168	. . . . A    
1169	. . . . T    
1170	. . . . T    
1171	. . . . T    
1172	. . . . T    
1173	. . . . A    
1174	. . . . C    
1175	. . . . T    
1176	. . . . A    
1177	. . . . C    
1178	. . . . G    
1179	. . . . T    
1180	. . . . G    
1181	. . . . T    
1182	. . . . C    
1183	. . . . C    
1184	. . . . A    
1185	. . . . A    
1186	. . . . A    
1187	. . . . G    
1188	. . . . A    
1189	. . . . T    
1190	. . . . C    
1191	. . . . A    
1192	. . . . A    
1193	. . . . C    
1194	. . . . A    
1195	. . . . G    
1196	. . . . A    
1197	. . . . G    
1198	. . . . A    
1199	. . . . T    
1200	. . . . G    
1201	. . . . A    
1202	. . . . A    
1203	. . . . G    
1204	. . . . C    
1205	. . . . A    
1206	. . . . C    
1207	. . . . T    
1208	. . . . T    
1209	. . . . T    
1210	. . . . A    
1211	. . . . G    
1212	. . . . G    
1213	. . . . G    
1214	. . . . T    
1215	. . . . G    
1216	. . . . G    
1217	. . . . T    
1218	. . . . C    
1219	. . . . C    
1220	. . . . T    
1221	. . . . C    
1222	. . . . C    
1223	. . . . T    
1224	. . . . G    
1225	. . . . T    
1226	. . . . T    
1227	. . . . G    
1228	. . . . T    
1229	. . . . C    
1230	. . . . C    
1231	. . . . T    
1232	. . . . G    
1233	. . . . T    
1234	. . . . G    
1235	. . . . G    
1236	. . . . T    
1237	. . . . A    
1238	. . . . T    
1239	. . . . G    
1240	. . . . T    
1241	. . . . C    
1242	. . . . C    
1243	. . . . T    
1244	. . . . A    
1245	. . . . T    
1246	. . . . T    
1247	. . . . C    
1248	. . . . A    
1249	. . . . C    
1250	. . . . T    
1251	. . . . G    
1252	. . . . T    
1253	. . . . G    
1254	. . . . G    
1255	. . . . T    
1256	. . . . A    
1257	. . . . T    
1258	. . . . G    
1259	. . . . T    
1260	. . . . T    
1261	. . . . G    
1262	. . . . A    
1263	. . . . C    
1264	. . . . T    
1265	. . . . G    
1266	. . . . T    
1267	. . . . G    
1268	. . . . T    
1269	. . . . T    
1270	. . . . T    
1271	. . . . C    
1272	. . . . C    
1273	. . . . A    
1274	. . . . T    
1275	. . . . T    
1276	. . . . A    
1277	. . . . A    
1278	. . . . A    
1279	. . . . A    
1280	. . . . C    
1281	. . . . T    
1282	. . . . G    
1283	. . . . A    
1284	. . . . T    
1285	. . . . C    
1286	. . . . T    
1287	. . . . T    
1288	. . . . A    
1289	. . . . C    
1290	. . . . A    
1291	. . . . T    
1292	. . . . C    
1293	. . . . C    
1294	. . . . A    
1295	. . . . A    
1296	. . . . C    
1297	. . . . A    
1298	. . . . G    
1299	. . . . A    
1300	. . . . A    
1301	. . . . G    
1302	. . . . T    
1303	. . . . A    
1304	. . . . C    
1305	. . . . C    
1306	. . . . T    
1307	. . . . T    
1308	. . . . A    
1309	. . . . A    
1310	. . . . G    
1311	. . . . C    
1312	. . . . A    
1313	. . . . G    
1314	. . . . A    
1315	. . . . G    
1316	. . . . A    
1317	. . . . A    
1318	. . . . A    
1319	. . . . A    
1320	. . . . G    
1321	. . . . T    
1322	. . . . G    
1323	. . . . G    
1324	. . . . G    
1325	. . . . A    
1326	. . . . C    
1327	. . . . A    
1328	. . . . C    
1329	. . . . C    
1330	. . . . T    
1331	. . . . C    
1332	. . . . A    
1333	. . . . C    
1334	. . . . C    
1335	. . . . A    
1336	. . . . T    
1337	. . . . A    
1338	. . . . C    
1339	. . . . A    
1340	. . . . G    
1341	. . . . T    
1342	. . . . G    
1343	. . . . A    
1344	. . . . A    
1345	. . . . T    
1346	. . . . T    
1347	. . . . G    
1348	. . . . A    
1349	. . . . T    
1350	. . . . C    
1351	. . . . A    
1352	. . . . T    
1353	. . . . C    
1354	. . . . A    
1355	. . . . C    
1356	. . . . C    
1357	. . . . A    
1358	. . . . G    
1359	. . . . A    
1360	. . . . C    
1361	. . . . C    
1362	. . . . T    
1363	. . . . G    
1364	. . . . G    
1365	. . . . G    
1366	. . . . T    
1367	. . . . T    
1368	. . . . C    
1369	. . . . A    
1370	. . . . A    
1371	. . . . A    
1372	. . . . T    
1373	. . . . A    
1374	. . . . C    
1375	. . . . A    
1376	. . . . A    
1377	. . . . G    
1378	. . . . T    
1379	. . . . A    
1380	. . . . T    
1381	. . . . T    
1382	. . . . T    
1383	. . . . T    
1384	. . . . A    
1385	. . . . T    
1386	. . . . T    
1387	. . . . A    
1388	. . . . T    
1389	. . . . T    
1390	. . . . T    
1391	. . . . G    
1392	. . . . T    
1393	. . . . T    
1394	. . . . C    
1395	. . . . T    
1396	. . . . T    
1397	. . . . A    
1398	. . . . T    
1399	. . . . T    
1400	. . . . T    
1401	. . . . T    
1402	. . . . C    
1403	. . . . A    
1404	. . . . A    
1405	. . . . A    
1406	. . . . T    
1407	. . . . A    
1408	. . . . A    
1409	. . . . T    
1410	. . . . G    
1411	. . . . T    
1412	. . . . G    
1413	. . . . G    
1414	. . . . C    
1415	. . . . C    
1416	. . . . T    
1417	. . . . A    
1418	. . . . A    
1419	. . . . G    
1420	. . . . C    
1421	. . . . A    
1422	. . . . A    
1423	. . . . T    
1424	. . . . T    
1425	. . . . A    
1426	. . . . C    
1427	. . . . T    
1428	. . . . T    
1429	. . . . A    
1430	. . . . T    
1431	. . . . T    
1432	. . . . T    
1433	. . . . T    
1434	. . . . G    
1435	. . . . G    
1436	. . . . A    
1437	. . . . A    
1438	. . . . G    
1439	. . . . T    
1440	. . . . A    
1441	. . . . A    
1442	. . . . T    
1443	. . . . T    
1444	. . . . T    
1445	. . . . A    
1446	. . . . A    
1447	. . . . A    
1448	. . . . A    
1449	. . . . G    
1450	. . . . T    
1451	. . . . A    
1452	. . . . T    
1453	. . . . T    
1454	. . . . T    
1455	. . . . T    
1456	. . . . C
```
